# Antinociceptive Analysis of Natural Monoterpenes Eugenol, Menthol, Carvacrol and Thymol in a Zebrafish Larval Model

**DOI:** 10.3390/ph17040457

**Published:** 2024-04-02

**Authors:** Cláudia Alexandra Rocha, Luís M. Félix, Sandra Mariza Monteiro, Carlos Venâncio

**Affiliations:** 1 School of Life and Environmental Sciences, University of Trás-os-Montes and Alto Douro (UTAD), 5000-801 Vila Real, Portugal; xana3@live.com.pt (C.A.R.); smonteir@utad.pt (S.M.M.); 2 Centre for the Research and Technology of Agro-Environment and Biological Sciences (CITAB), University of Trás-os-Montes and Alto Douro (UTAD), 5000-801 Vila Real, Portugal; 3 Institute for Innovation, Capacity Building and Sustainability of Agri-Food Production (Inov4Agro), University of Trás-os-Montes and Alto Douro (UTAD), 5000-801 Vila Real, Portugal; 4 Department of Animal Science, School of Agrarian and Veterinary Sciences, University of Trás-os-Montes and Alto Douro (UTAD), 5000-801 Vila Real, Portugal; 5 Veterinary and Animal Research Centre (CECAV), Associate Laboratory of Animal and Veterinary Sciences (AL4AnimalS), University of Trás-os-Montes and Alto Douro (UTAD), 5000-801 Vila Real, Portugal

**Keywords:** antinociception, analgesia, monoterpenes, zebrafish, eugenol, menthol, carvacrol, thymol

## Abstract

In the last decade, a considerable number of studies have broadened our knowledge of the nociceptive mechanisms of pain, a global health problem in both humans and animals. The use of herbal compounds such as eugenol, menthol, thymol, and carvacrol as analgesic agents has accompanied the growing interest in this area, offering a possible solution for this complex problem. Here, we aimed to explore how these natural substances—at three different concentrations (2, 5 and 10 mg/L)—affect the pain responses in zebrafish (*Danio rerio*) larvae exposed to 0.05% acetic acid (AA) for 1 min. By analysing the activity of acetylcholinesterase (AChE), 5′-ectonucleotidase and NTPDases, as well as aversion and exploratory behaviours, it was observed that that although all substances were effective in counteracting the pain stimulus, the concentration range within which they do so might be very limited. Eugenol, despite its acknowledged properties in fish anaesthesia, failed to alleviate the pain stimulus at low concentrations. Contrastingly, menthol exhibited the most promising results at the lowest concentrations tested. Overall, it is concluded that menthol might be a good analgesic for this species, qualifying it as a substance of interest for prospective studies.

## 1. Introduction

Pain is a global health problem for humans and animals, constituting a perpetual obstacle in the pursuit of well-being [1,2]. Even though mammals exhibit a higher level of brain development, the nociceptive pain mechanism is well conserved across animals [3,4]. In the last decade, the number of studies enriching our understanding of nociceptive responses in fish has increased, reinforcing the need to find analgesic compounds for these animals, which are currently scarce [3,5]. Furthermore, this knowledge makes it possible to use fish as nociceptive models to screen new analgesic compounds and their properties [6,7].

Advancements in natural products have allowed the development of new therapeutic solutions leveraging their potential bioactive properties, such as analgesic compounds [8]. Eugenol, a main constituent of clove oil, stands out as one of the most common plant-based anaesthetics in fish owing to its proven safety for aquatic life and the surrounding environment [9,10]. Despite this, concerns persist regarding its effectiveness as an analgesic in fish [11,12]. Menthol, a cyclic monoterpene alcohol, is a major constituent of the essential oils of Mentha (*Lamiaceae*) species [13]. The versatility of this substance is undeniable, as it is frequently used as a food additive, in addition to cosmetics, insecticides, and it is even employed for therapeutic purposes [13,14]. Although menthol has been demonstrated to be an effective anaesthetic in several fish species, its efficacy as an analgesic seems to be contingent upon concentration [9,13,14]. As outlined in the review by Li and Zhang [14], menthol exhibits distinct analgesic effects: low to moderate concentrations induce a sensation of cooling, while high concentrations evoke cold allodynia and cold hyperalgesia [14]. In addition, menthol activates the transient receptor potential cation channel subfamily A member 1 (TRPA1), leading to irritation effects [14]. Thymol and carvacrol are isomers of monoterpenic phenols with similar effects, acting on different nociceptive receptors [15]. The analgesic effect of carvacrol seems to be mediated by descending pain-inhibitory mechanisms due to its anti-inflammatory properties, and it may also interact with the opioid system [16]. Similarly, thymol has many pharmacological properties, from which its antinociceptive activity is highlighted [17,18]. However, as far as we know, fish have not yet sufficiently explored these properties.

Nociception, the process through which the nervous system detects and processes harmful stimuli, relies on the collective action of many enzymes, such as acetylcholinesterase (AChE) and ectonucleotidases (ecto-5′-ncleotidases and NTP-dases) [6,19,20,21,22]. AChE is responsible for terminating cholinergic neurotransmission by hydrolysing acetylcholine, a neurotransmitter that influences pain perception [20,21]. Simultaneously, ectonucleotidases regulate extracellular levels of adenine nucleotides (AMP, ADP, and ATP) implicated in various physiological processes associated with pain modulation [19,21]. In addition, ectonucleotidases are responsible for a hydrolysis cascade that leads to adenosine formation, which acts on adenosine receptors in nociceptive neurons [19,21]. Understanding the activities of both these enzymes becomes imperative for unravelling the mechanisms underlying antinociception and exploring potential therapeutic targets for pain management.

The zebrafish (*Danio rerio*) larval model has been established as an excellent alternative for studying pain perception due to its proven similarity with mammals’ biochemical and physiological processes integrated into this species’ behavioural responses [6,23,24,25]. Moreover, the nociceptive pain responses were confirmed in zebrafish larvae through exposure to nociceptive agents, such as acetic acid (AA), resulting in behavioural and biochemical alterations [26,27]. Therefore, this study evaluated the analgesic properties of active monoterpenes, such as eugenol, menthol, carvacrol, and thymol, assessing biochemical and behavioural endpoints.

## 2. Results

### 2.1. Exploratory and Aversive Behaviour

#### 2.1.1. Eugenol

**Figure 1:**
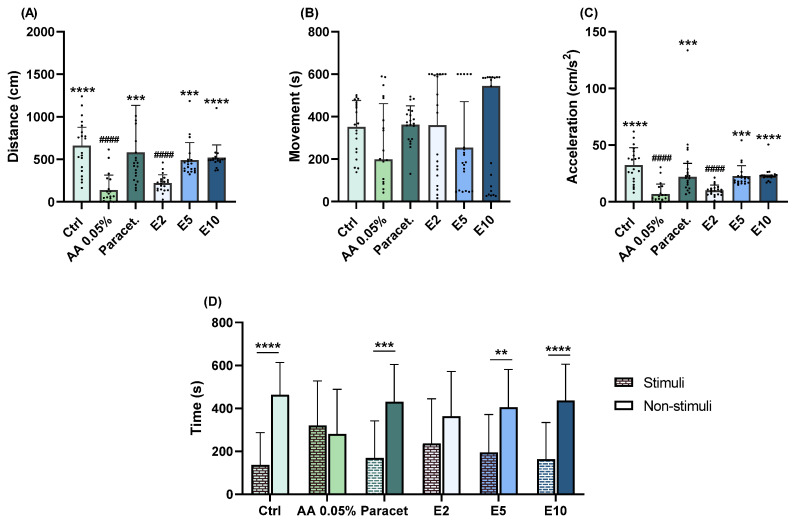
Assessment of exploratory (distance covered (**A**), movement (**B**), acceleration (**C**)) and aversive (**D**) behaviours of zebrafish larvae exposed to 2 (E2), 5 (E5), and 10 (E10) mg/L of eugenol followed by a 1 min exposure to 0.05% AA. Statistical analysis for the exploratory behaviour parameters was performed using the Kruskal–Wallis test followed by Dunn’s pairwise multiple comparison test. Data are presented as mean ± interquartile range. Statistical analysis for the aversive behaviour parameters was performed using the *t*-test, and data are presented as mean ± standard deviation (SD). * indicates significant differences to the AA group (** *p* ≤ 0.01, *** *p* ≤ 0.001, and **** *p* ≤ 0.0001). # indicates significant differences to the control group (#### *p* ≤ 0.0001).

At a concentration of 2 mg/L, eugenol induced a significant decrease in distance covered (*p* < 0.0001; Figure 1A) and acceleration (*p* < 0.0001; Figure 1C) compared to the control group. At concentrations of 5 and 10 mg/L, eugenol consistently affected exploratory behaviour compared to the AA group. Larvae exposed to these concentrations exhibited a significant increase in distances covered (E5: *p* = 0.0002; E10: *p* < 0.0001; Figure 1A) and acceleration (E5: *p* = 0.0004; E10: *p* < 0.0001; Figure 1C). Additionally, regarding aversive behaviour, significant differences between stimuli and non-stimuli were only observed at concentrations of 5 (*p* = 0.0076; Figure 1D) and 10 (*p* < 0.0001; Figure 1D) mg/L.

#### 2.1.2. Menthol

**Figure 2:**
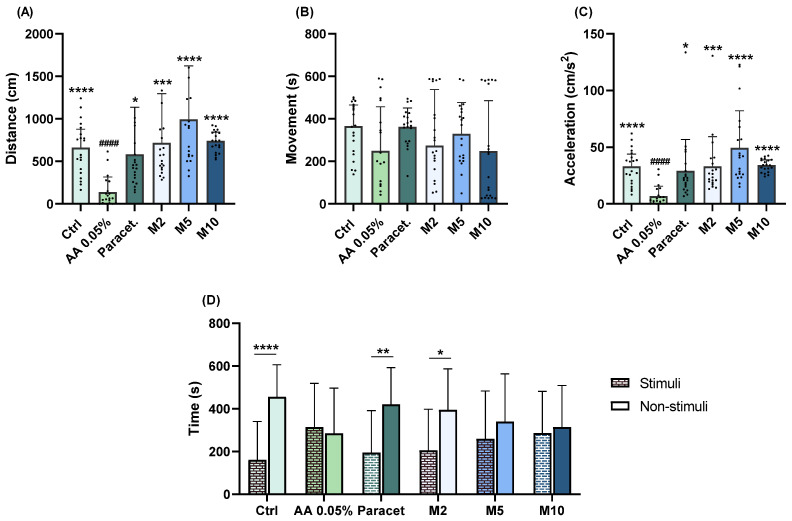
Assessment of exploratory (distance covered (**A**), movement (**B**), acceleration (**C**)) and aversive (**D**) behaviours of zebrafish larvae exposed to 2 (M2), 5 (M5), and 10 (M10) mg/L of menthol followed by a 1 min exposure to 0.05% AA. Statistical analysis for the exploratory behaviour parameters was performed using the Kruskal–Wallis test followed by Dunn’s pairwise multiple comparison test. Data are presented as mean ± interquartile range. Statistical analysis for the aversive behaviour parameters was performed using the *t*-test, and data are presented as mean ± standard deviation (SD). * indicates significant differences to the AA group (* *p* ≤ 0.05, ** *p* ≤ 0.01, *** *p* ≤ 0.001, and **** *p* ≤ 0.0001). # indicates significant differences to the control group (#### *p* ≤ 0.0001).

In terms of exploratory behaviour, each menthol concentration yielded similar results compared to the AA group. Larvae exposed to a concentration of 2 mg/L exhibited a significantly higher distance covered (*p* = 0.0002; Figure 2A) and acceleration (*p* = 0.0005; Figure 2C). Similarly, at 5 mg/L, the larvae demonstrated a significantly higher distance covered (*p* < 0.0001; Figure 2A) and acceleration (*p* < 0.0001; Figure 2C). At 10 mg/L, similar changes were observed in both distances covered (*p* < 0.0001; Figure 2A) and acceleration (*p* < 0.0001; Figure 2C). Notably, in terms of aversive behaviour, significant differences between stimuli and non-stimuli (*p* = 0.406; Figure 2D) were only observed at a concentration of 2 mg/L.

#### 2.1.3. Carvacrol

**Figure 3:**
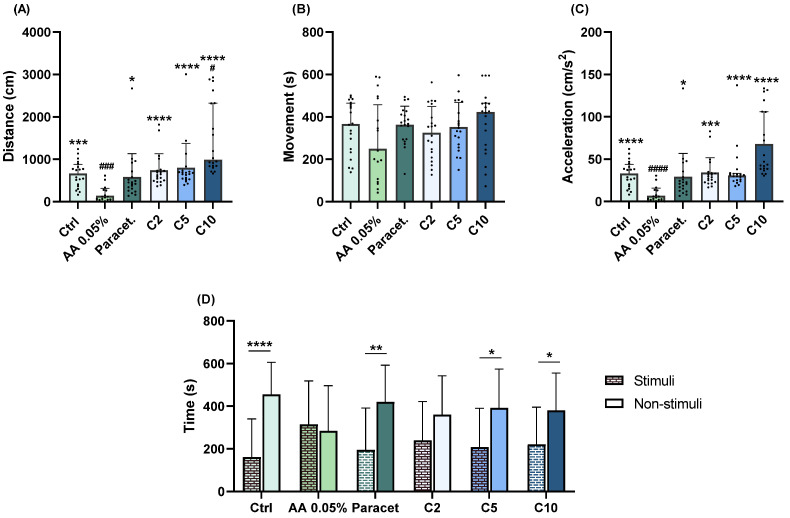
Assessment of exploratory (distance covered (**A**), movement (**B**), acceleration (**C**)) and aversive (**D**) behaviours of zebrafish larvae exposed to 2 (C2), 5 (C5), and 10 (C10) mg/L of carvacrol followed by a 1 min exposure to 0.05% AA. Statistical analysis for the exploratory behaviour parameters was performed using the Kruskal–Wallis test followed by Dunn’s pairwise multiple comparison test. Data are presented as mean ± interquartile range. Statistical analysis for the aversive behaviour parameters was performed using the *t*-test, and data are presented as mean ± standard deviation (SD). * indicates significant differences to the AA group (* *p* ≤ 0.05, ** *p* ≤ 0.01, *** *p* ≤ 0.001, and **** *p* ≤ 0.0001). # indicates significant differences to the control group (# *p* ≤ 0.05, ### *p* ≤ 0.001, and #### *p* ≤ 0.0001).

Each concentration of carvacrol demonstrated analogous results when compared to the AA group. At 2 mg/L, significantly higher values for distance covered (*p* < 0.0001; Figure 3A) and acceleration (*p* =0.0001; Figure 3C) were observed. Similarly, at 5 mg/L, there were significant increases in both distance covered (*p* < 0.0001; Figure 3A) and acceleration (*p* < 0.0001; Figure 3C). At 10 mg/L, significantly higher values for both distance covered (*p* < 0.0001; Figure 3A) and acceleration (*p* < 0.0001; Figure 3C) were also observed. In comparison to the control group, animals exposed to the higher concentration (10 mg/L) exhibited significant differences in distance covered (*p* = 0.0216; Figure 3A). In aversive behaviour, significant differences between stimuli and non-stimuli were observed only at 5 (*p* = 0.014; Figure 3D) and 10 (*p* = 0.0488; Figure 3D) mg/L.

#### 2.1.4. Thymol

**Figure 4:**
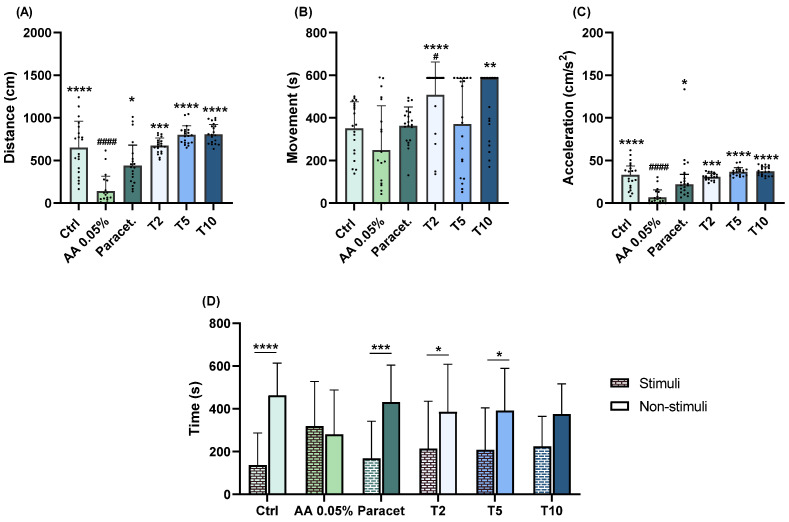
Assessment of exploratory (distance covered (**A**), movement (**B**), acceleration (**C**)) and aversive (**D**) behaviours of zebrafish larvae exposed to 2 (T2), 5 (T5), and 10 (T10) mg/L of thymol followed by a 1 min exposure to 0.05% AA. Statistical analysis for the exploratory behaviour parameters was performed using the Kruskal–Wallis test followed by Dunn’s pairwise multiple comparison test. Data are presented as mean ± interquartile range. Statistical analysis for the aversive behaviour parameters was performed using the *t*-test, and data are presented as mean ± standard deviation (SD). * indicates significant differences to the AA group (* *p* ≤ 0.05, ** *p* ≤ 0.01, *** *p* ≤ 0.001, and **** *p* ≤ 0.0001). # indicates significant differences to the control group (# *p* ≤ 0.05 and #### *p* ≤ 0.0001).

**Figure 5:**
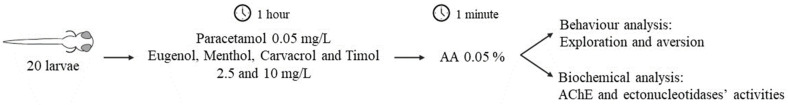
Workflow of the drug exposure procedure.

At a concentration of 2 mg/L, thymol differs from the AA group in both distance covered (*p* = 0.0002; Figure 4A) and acceleration (*p* = 0.0004; Figure 4C). However, at concentrations of 5 and 10 mg/L, thymol demonstrated identical results to the AA group in both distance covered (*p* < 0.0001; Figure 4A) and acceleration (*p* < 0.0001; Figure 4B). At 2 mg/L, significantly increased movement was also observed compared to both the control (*p* = 0.0178; Figure 4B) and AA (*p* < 0.0001; Figure 4B) groups. Additionally, at 10 mg/L, significant increases in movement (*p* = 0.0048; Figure 4B) were noted compared to the AA group. Regarding aversive behaviour, significant differences between stimuli and non-stimuli were observed only at 2 (*p* = 0.0275; Figure 4D) and 5 (*p* = 0.0112; Figure 4D) mg/L.

### 2.2. Acetylcholinesterase (AChE) Activity

The tested substances did not induce significant alterations (*p* > 0.05) in AChE activity (Table 1) at any concentration.

### 2.3. 5′-Ectonucleotidase (AMP Hydrolysis) and NTPDase (ADP and ATP Hydrzolysis) Activities

The substances used revealed no significant alterations (*p* > 0.05) in the ectonucleotidases activities (Table 1).

## 3. Discussion

In recent years, there has been a growing recognition that many procedures are potentially painful for fish, yet there remains a scarcity of analgesics tested and approved for use in this animal class [5]. Essential oils present in many plants are known to have analgesic properties, although some monoterpenes such as eugenol, menthol, carvacrol, and thymol have not been explored for this end, warranting further investigation [28]. This study examined potential behaviour and biochemical alterations in zebrafish larvae after exposure to these natural compounds, followed by immersion in a known pain-inducing agent, acetic acid (AA).

The results of this study show a disruption of the behavioural outcomes caused by AA. The nociceptive impact of a 0.05% AA solution on zebrafish larvae is unmistakable, evidenced by a reduction in the normal exploratory behaviour across all specimens. Typically, when introduced in novel conditions, larvae tend to fiercely investigate their surroundings until they acclimate, which is reflected in their locomotor activity [26]. However, immersion in AA alters this consistent behavioural response, strongly suggesting the induction of a painful stimulus and corroborating previous research [27,29,30,31]. In contrast, when paracetamol was tested, no alterations in behavioural parameters were observed, confirming its well-established analgesic properties [32].

Regarding the tested monoterpenes, all compounds have acknowledged anaesthetic properties when administered at their specific anaesthetic doses [18,33,34]. In this study, we observed that all induced different behavioural responses to those of the AA group, hinting at their analgesic potential when administered at lower concentrations. The lowest eugenol concentration drifts from this pattern, inducing no behavioural alterations compared to the AA group, thus highlighting a possible concentration-dependent influence on the behaviour of zebrafish larvae. Even though eugenol stands as the most extensively studied fish anaesthetic, our findings verify its inefficacy in countering the pain stimulus induced by AA at lower concentrations, as previously shown [35,36].

Among the substances tested, only menthol is officially recognised as an analgesic [15,37]. Interestingly, despite all menthol concentrations showing similar results on exploratory behaviour, only the lowest concentration seemed to allow for recognition of aversive stimuli. Moreover, the larvae seemed to reduce exploration under the influence of higher concentrations. Previous research on rainbow trout (*Oncorhynchus mykiss*) revealed menthol’s sedative effects at concentrations ranging from 10 to 20 mg/L [38]. The efficacy of each concentration depends on the size and species of the animal, and because these relatively low concentrations induce sedation in this larger species, it hints that our chosen concentrations for zebrafish may have a similar effect [39]. Furthermore, because sedation is characterised as a state of drowsiness and reduced sensory perception, it could explain the decreased responsiveness to aversive stimuli observed under the effect of higher concentrations [40]. Menthol’s analgesic effect relies on activation of ionotropic receptors belonging to the transient receptor potential family, such as TRPM8 and TRPA1, with only the latter present in the zebrafish genome [14,15,41]. TRPA1 is instrumental in sensing harmful chemical stimuli in zebrafish, consequently playing a vital role in the nociception process and avoidance behaviours [41,42]. The involvement of TRPA1 in pain pathways suggests that it could be a promising target for developing analgesic therapies, which in turn could include substances that interact with this type of receptor, such as menthol. Nevertheless, since our study found the most promising effects only at 2 and 5 mg/L, it is prudent to assume that this remarkable substance may demonstrate its analgesic effect within a short range of experimental concentrations. This underscores the need for further research to determine the best concentration for menthol’s pain-relieving effects.

Thymol and carvacrol are identical substances with distinct mechanisms influencing pain perception [43]. Carvacrol, identical to menthol, interacts with TRPA1 receptors, while thymol activates the TRPV3 receptor responsible for sensing noxious heat [43]. These two substances have different side effects depending on the administered concentration [13,44]. Evidence in silver catfish (*Rhamdia quelen*) shows that thymol and carvacrol induce sedation and muscle contractions at concentrations as low as 25 mg/L, with the latter being responsible for more pronounced contractions and a longer recovery time [13,44]. Despite these effects, at lower concentrations, carvacrol is considered one of the safest substances due to its protective properties [45]. At a concentration of 2 and 5 mg/L, both thymol and carvacrol exhibited promising results, demonstrating potential analgesic effects without inducing any adverse effects on the larval behaviour. Thymol has been hypothesised to have higher efficacy in common carp (*Cyprinus carpio*), than other natural anaesthetic agents such as eugenol and menthol [46]. As such, its analgesic potential might also be similar. Nevertheless, larvae exposed to the higher concentration of thymol failed to recognise and avoid the aversive stimulus. Similarly, larvae exposed to 10 mg/L of carvacrol showed a significant increase in all locomotion parameters, suggesting that the stress induced by AA may not have been alleviated. The studied monoterpenes have low molecular weight and high lipid solubility, which makes their absorption and distribution quick [47,48]. Despite this, the elimination of these substances takes longer, a fact that may explain our results [47,48]. These outcomes may also be attributed to the naturally low sedative concentrations of the substances, and the narrow range of experimental analgesic concentrations [13,44].

The AchE activity was also assessed due to its relationship with behavioural outcomes and its role as a neurotoxicity biomarker [49]. AchE is responsible for hydrolysing acetylcholine (Ach) and terminating cholinergic activity, thus regulating cholinergic neurotransmission [50,51]. When AchE is inhibited, Ach accumulates in the synaptic cleft, leading to prolonged stimulation of cholinergic neurons, which can result in neurotoxic effects, as well as depressive and anxiety-like behaviours that reflect on the exploratory behaviour [49,50,51]. Contrastingly, because Ach is a powerful neurotransmitter that acts on peripheral antinociception, inhibition of AchE is also intimately connected with pain modulation and might offer beneficial effects under controlled situations [19,20,52,53,54,55]. Eugenol, menthol, thymol, and carvacrol have all been identified as potential inhibitors of AChE activity when administered at appropriate concentrations [56,57,58]. In addition to the behavioural evidence, we observed no changes in the activity of this enzyme, suggesting that substances’ exposure concentrations may have been below the threshold required to inhibit AChE activity and influence pain perception.

To investigate the effects of the chemical stimulus induced by AA on the levels of AMP, ADP, and ATP, the activities of ecto-5′-nucleotidase and NTPDases, enzymes responsible for hydrolysing these nucleotides, were measured [19,21]. This process holds significant importance, as these nucleotides are fundamental in diverse physiological processes that interfere with pain perception [21]. ATP—released by cells under unusual stress conditions, such as changes in pH—is responsible for activating purinergic receptors on sensory nerve terminals, initiating nociceptive signalling [59,60]. On the other hand, adenosine, the final product of the hydrolysis cascade, is well-recognised for its antinociceptive properties due to its relationship with adenosine receptors expressed in nociceptive neurons, spinal cord neurons, and other cells [19,60,61]. In situations of repeated stress, the activity of ectonucleotidases is often altered, consequently influencing AMP, ADP, and ATP levels and antinociceptive mechanics [21,59,62]. Many studies suggest that an increase in AMP hydrolysis, and consequent adenosine generation, is often essential to how AA modulates ectonucleotidase activities [26,60,63,64]. In situations of repeated stress, there is evidence of a decreased AMP hydrolysis, which influences adenosine formation and justifies hypernociception [64]. Adenosine synthesis in adequate quantities mainly occurs when both ATP and ADP levels are decreased because these nucleotides inhibit CD73, the 5′-ectonucleotidase responsible for AMP conversion [26,64,65]. Still, AMP hydrolysis is a complex process that might involve more than one enzymatic agent; in CD73 knockout mice, AMP hydrolysis still occurs, and it is less evident in lower pH environments [61,65]. Despite our study’s similarities with previous research, we must consider that numerous factors contribute to the complex nature of pain mechanisms, and that the relationship between ectonucleotidases and pain may be more complex than previously understood. Our findings emphasise the need for future investigation that will eventually uncover the nuances of how AA impacts the activity of these enzymes, as well as the larger picture of pain perception.

## 4. Materials and Methods

### 4.1. Zebrafish Maintenance

Wild-type zebrafish (Danio rerio, AB strain) were maintained at a temperature of 28 ± 1 °C in a room with a 14:10 h light:dark cycle, within an open water system equipped with mechanical and biological filtration [66]. Adults were grouped in tanks in a ratio of 2 males to 1 female to produce embryos, and spawning was induced in the morning at the start of the light period [66]. Newly fertilized eggs underwent a thorough cleaning process (immersion in 0.5% chloramine T, and a double wash with E3 buffer) [66]. The eggs were then randomly distributed in plates until they reached 5 days post-fertilization (dpf), with daily changes in E3 buffer (5 mM NaCl, 0.17 mM KCl, 0.33 mM CaCl_2_, and 0.33 mM MgSO_4_ (pH 7.2–7.4) [67]. The deceased fish were also removed daily. The ethical guidelines of the European (European Directive, 2010/63) and Portuguese (Decreto-Lei 113/2013) law governing the protection of animals used for scientific purposes were followed throughout all experimental procedures and sample collection.

### 4.2. Drug Exposure

In 24-well plates, 5 dpf larvae were individually exposed to four natural compounds—Eugenol 99% (CAS number: 97-53-0; Alfa Aesar, Oeiras, Portugal), Thymol 100% (CAS number: 89-83-8; Merck Millipore, Algés, Portugal), Menthol 98% (CAS number: 2216-51-5; Alfa Aesar, Oeiras, Portugal), and Carvacrol 98% (CAS number: 499-75-2; London, UK)—at three different concentrations (2, 5, and 10 mg/L), for a duration of 1 h (Figure 5) [26]. These concentrations were selected based on the substances 10-times higher anaesthetic doses [68] and prepared by diluting a previously made stock solution of 89 g/L for each substance (menthol and eugenol, pH 7.2 ± 0.1 and 7.1 ± 0.1, respectively) in 90% alcohol [69]. After exposure, the larvae were removed and subjected to a 1 min exposure to 0.05% AA (CAS number: 64-19-7; Fisher Scientific, Oeiras, Portugal) diluted with E3 buffer, to induce pain [26]. To ensure this method works, a positive control using paracetamol (CAS number: 103-90-2; extracted from tablets using propanone and dissolved in E3 buffer) at a concentration of 0.05 mg/L was also evaluated [27,70].

### 4.3. Behaviour Assessment

#### 4.3.1. Exploratory Behaviour

Following exposure, 20 zebrafish larvae from each compound’s concentration were separately placed into each well of a 12-well plate, each with an agarose ring and filled with E3 buffer [71]. After 5 min of acclimatation, the larvae were filmed using a digital camera for 10 min at an approximate distance of 50 cm, following an established protocol by Gusso and Cruz [27,71]. The recorded data were evaluated in TheRealFishTracker software (Version 0.4.0; Dynamic Graphics Project; University of Toronto Mississauga, Mississauga, ON, Canada), and the larvae behaviour was assessed by calculating the amount of time the larvae were moving (s), acceleration (cm/s^2^) and distance covered (m) [71].

#### 4.3.2. Aversive Behaviour

After exposure, 20 zebrafish larvae from each compound’s concentration were separately placed in 12-well plates filled with E3 buffer, according to the protocol described in Felix and Antunes [71]. Following a 5 min acclimatation period, the larvae were exposed to a recognized visual aversive stimulus—a red bouncing ball (1.35 cm diameter) moving left–right–left at a speed of 1 cm/s over a straight 2 cm trajectory on the top half of the well—for 10 min [72]. The red bouncing ball (Red–Green–Blue (RGB) values: 255, 0, 0) against the white background (RGB: 255, 255, 255) was part of an animated presentation created with Microsoft PowerPoint (Version 2304). The videos were manually analysed, and the percentage of time spent in each zone of the well was registered in Microsoft Excel (Version 2304).

### 4.4. Biochemical Analysis

#### 4.4.1. AChE Activity

Based on the analgesia effects observed in the behavioural tests, thirty larvae from five new exposed replicates to each compound’s intermediate concentration (5 mg/L) were collected in ice-cold homogenization buffer (0.32 M Sucrose, 0.1 mM EDTA, 5 M HEPES, pH 7.5), and the samples were homogenized in a biological sample lyser (TissueLyserII, QIAGEN, Hilden, Germany) at 30 Hz for 1 min and 30 s. Then, the sample was centrifuged at 4 °C for 10 min at 12,000× *g*, and the remaining supernatant was collected and used to quantify the total protein at 280 nm using a Power Wave XS2 microplate reader (Bio-Tek Instruments, Winooski, VT, USA). Later, 10 µL of these samples were mixed with 180 µL of 5,5′-dithiobis(2 nitrobenzoic acid) (DTNB) (0.05 mM) in 0.05 M Tris Buffer (pH 7.4), according to the protocol described by Rodriguez-Fuentes and Rubio-Escalante [73]. To start the reaction, 10 µL of acetylthiocholine iodide (20 mM) was added. Using a Power Wave XS2 microplate reader (Bio-Tek Instruments, USA), AChE activity was measured according to the absorbance readings at 405 nm, and calculated assuming the absorption coefficient of 13.60 mM/cm. The final data were expressed as nmol of TNB oxidized per min and mg protein (nmol TNB/min·mg protein).

#### 4.4.2. 5′-Ectonucleotidase (AMP Hydrolysis) and NTPDase (ADP and ATP Hydrolysis) Activities

Thirty larvae from five new replicate exposures to each compounds’ intermediate concentration of 5 mg/L were collected following an adapted methodology described by Gusso and Wiprich [26], Veeramachineni, Ubayawardhana, and Murkin [74], and Kodama, Fukui, and Kometani [75]. The larvae were collected in 200 µL of tris-citrate buffer (50 mM Tris, 2 mM EDTA, 2 mM EGTA, pH 7.4) and the samples were centrifuged at 4 °C for 10 min at 800× *g*. The resulting supernatant was collected and subjected to a second centrifugation at 4 °C for 10 min at 21,000× *g*. The remaining pellets were then frozen at −80 °C, thawed, resuspended in 200 µL of tris-citrate buffer, and used for total protein quantification at 280 nm using a Power Wave XS2 microplate reader (Bio-Tek Instruments, USA). To each 10 µL of original sample or 10× diluted (NTPDase activities), 180 µL of tris-HCl (50 mM) containing 5 mM of CaCl_2_ (NTPDase activities) or 5 mM MgCl_2_ (ecto-5′-nucleotidase activity) was added. The mixtures were incubated at 37 °C for 10 min before adding the respective substrates, AMP, ADP, and ATP (1 mM), to initialize the reaction. After 30 min at 37 °C, the reaction was halted by adding 50 µL of the colour reagent (0.055% (*w*/*v*) malachite green, 1.50% (*w*/*v*) ammonium molybdate tetrahydrate, 0.175% (*v*/*v*) polysorbate 20) [73]. After 5 min, the released Pi was quantified by measuring the samples’ absorbances at 630 nm using a Power Wave XS2 microplate reader (Bio-Tek Instruments, USA). The results were quantified using NaHPO4 (0–3.35 mM) as a Pi standard and the final data were expressed as mmol Pi per mg protein (mmol Pi/mg protein).

### 4.5. Statistical Analysis

Statistical analysis was performed using GraphPad Prism 9 for Windows (Version 9.5.0; La Jolla, CA, USA). For the remaining data, the normal distribution and homogeneity were evaluated by the Shapiro–Wilk test and the Brown–Forsythe test, respectively. The non-normally distributed data were assessed using the Kruskal–Wallis non-parametric test, followed by Dunn’s pairwise comparison tests, with the results presented as median and interquartile range. For normally distributed data, one-way analysis of variance (ANOVA), followed by Tukey’s pairwise comparison tests were performed, and results are presented as mean ± standard deviation (SD). The dependent sample Student’s *t* test was used to compare the differences between an animal behaviour when an aversive stimulus is presented or not. All statistical tests considered differences to be significant at *p* < 0.05.

## 5. Conclusions

Overall, our research indicates that eugenol, menthol, thymol, and carvacrol have the potential to influence pain perception without exhibiting severe consequences. However, their analgesic effects might only be observed within a limited range of experimental low concentrations. Despite being an acknowledged anaesthetic, eugenol failed to attenuate the pain stimulus at lower concentrations. Nevertheless, menthol demonstrated the most favourable results at the lowest concentrations tested, making it a promising substance to focus on.

## Figures and Tables

**Table 1:** AChE and ectonucleotidases (measured through AMP, ADP, and ATP hydrolysis) activities (mean ± SD) in zebrafish larvae exposed to 5 mg/L of eugenol, menthol, thymol, and carvacrol.

Sample ^1^	AChE	AMP	ADP	ATP
	nmol NADH/min·mg Protein	mmol Pi/mg Protein	mmol Pi/mg Protein	mmol Pi/mg Protein
Control	168.61 ± 34.39	2.82 ± 1.21	7.67 ± 0.99	73.73 ± 18.78
Acetic Acid	122.65 ± 15.04	1.99 ± 0.69	6.57 ± 1.91	71.53 ± 19.75
Paracetamol	144.26 ± 24.72	2.65 ± 0.93	6.89 ± 1.70	74.33 ± 16.67
Eugenol	112.21 ± 34.37	2.79 ± 0.45	8.70 ± 1.65	89.83 ± 12.89
Menthol	109.29 ± 20.36	3.60 ± 0.75	7.58 ± 1.45	86.18 ± 12.06
Thymol	141.71 ± 17.97	3.05 ± 0.88	8.17 ± 0.69	81.03 ± 14.90
Carvacrol	148.83 ± 14.63	3.07 ± 0.42	9.06 ± 5.73	81.61 ± 14.58

^1^ Each sample was tested with 5 replicas containing 30 larvae.

## Data Availability

The data supporting the conclusions of this article will be provided by the authors upon request.
